# Conservative management of a dislodged leadless pacemaker wedged in the pulmonary artery assessed by pulmonary perfusion scintigraphy: a case report

**DOI:** 10.1093/ehjcr/ytag749

**Published:** 2026-10-16

**Authors:** Takanori Arimoto, Daisuke Kutsuzawa, Naoaki Hashimoto, Ken Watanabe, Masafumi Watanabe

**Affiliations:** The Department of Cardiology, Pulmonology, and Nephrology, Yamagata University School of Medicine, 2-2-2 Iida-Nishi, Yamagata 990-9585, Japan; The Department of Cardiology, Pulmonology, and Nephrology, Yamagata University School of Medicine, 2-2-2 Iida-Nishi, Yamagata 990-9585, Japan; The Department of Cardiology, Pulmonology, and Nephrology, Yamagata University School of Medicine, 2-2-2 Iida-Nishi, Yamagata 990-9585, Japan; The Department of Cardiology, Pulmonology, and Nephrology, Yamagata University School of Medicine, 2-2-2 Iida-Nishi, Yamagata 990-9585, Japan; The Department of Cardiology, Pulmonology, and Nephrology, Yamagata University School of Medicine, 2-2-2 Iida-Nishi, Yamagata 990-9585, Japan

**Keywords:** Leadless pacemaker, Micra, Pulmonary artery embolization, Lung perfusion scintigraphy, Case report

## Abstract

**Background:**

Leadless pacemakers reduce complications associated with conventional transvenous systems; however, device dislodgement with pulmonary artery embolization is a rare complication unique to leadless pacemakers. Although prompt retrieval is generally recommended, retrieval from the pulmonary artery may carry procedural risks, particularly in elderly or high-risk patients.

**Case summary:**

An 84-year-old woman with complete atrioventricular block underwent implantation of a leadless pacemaker. Although the pacing threshold was favourable, the R-wave amplitude was low, and unstable device movement was observed after tether release, suggesting inadequate fixation. Two hours after implantation, pacing failure occurred, and chest radiography showed migration of the device into the right pulmonary artery. The following day, the device migrated to the left posterior basal segmental pulmonary artery and remained stably wedged. Because of the risk associated with retrieval, conservative management was selected, and a transvenous pacemaker was implanted. Serial lung perfusion scintigraphy demonstrated right-to-left perfusion distributions of 60.5% vs. 39.5% at 10 days, 59.1% vs. 40.9% at 3 months, and 58.5% vs. 41.5% at 1 year, without progressive deterioration in left lung volume. The patient remained asymptomatic over 3 years of follow-up.

**Discussion:**

This case illustrates that conservative management may be feasible in carefully selected high-risk patients when an embolized leadless pacemaker is stably wedged within the pulmonary artery. Serial pulmonary perfusion scintigraphy may provide objective evidence of preserved pulmonary perfusion and support long-term follow-up after conservative management.

Learning points

- Conservative management may be considered when an embolized leadless pacemaker is stably wedged in the pulmonary artery and retrieval risk is high.
- Serial lung perfusion scintigraphy can confirm preserved pulmonary perfusion and support long-term follow-up.

## Introduction

Leadless pacemakers were developed to overcome complications associated with conventional transvenous permanent pacemakers. Large real-world registries have demonstrated favourable long-term safety outcomes, with significantly lower rates of major complications compared with transvenous systems. Although device dislodgement is rare, pulmonary artery embolization represents a complication unique to leadless pacemakers.^1^ In such cases, prompt retrieval is generally recommended; however, retrieval from the pulmonary artery carries potential risks, including vascular injury and interference with intracardiac structures.^2,3^ The optimal management strategy for embolized leadless pacemakers, particularly in high-risk elderly patients, remains uncertain.

## Summary figure

**Figure:**
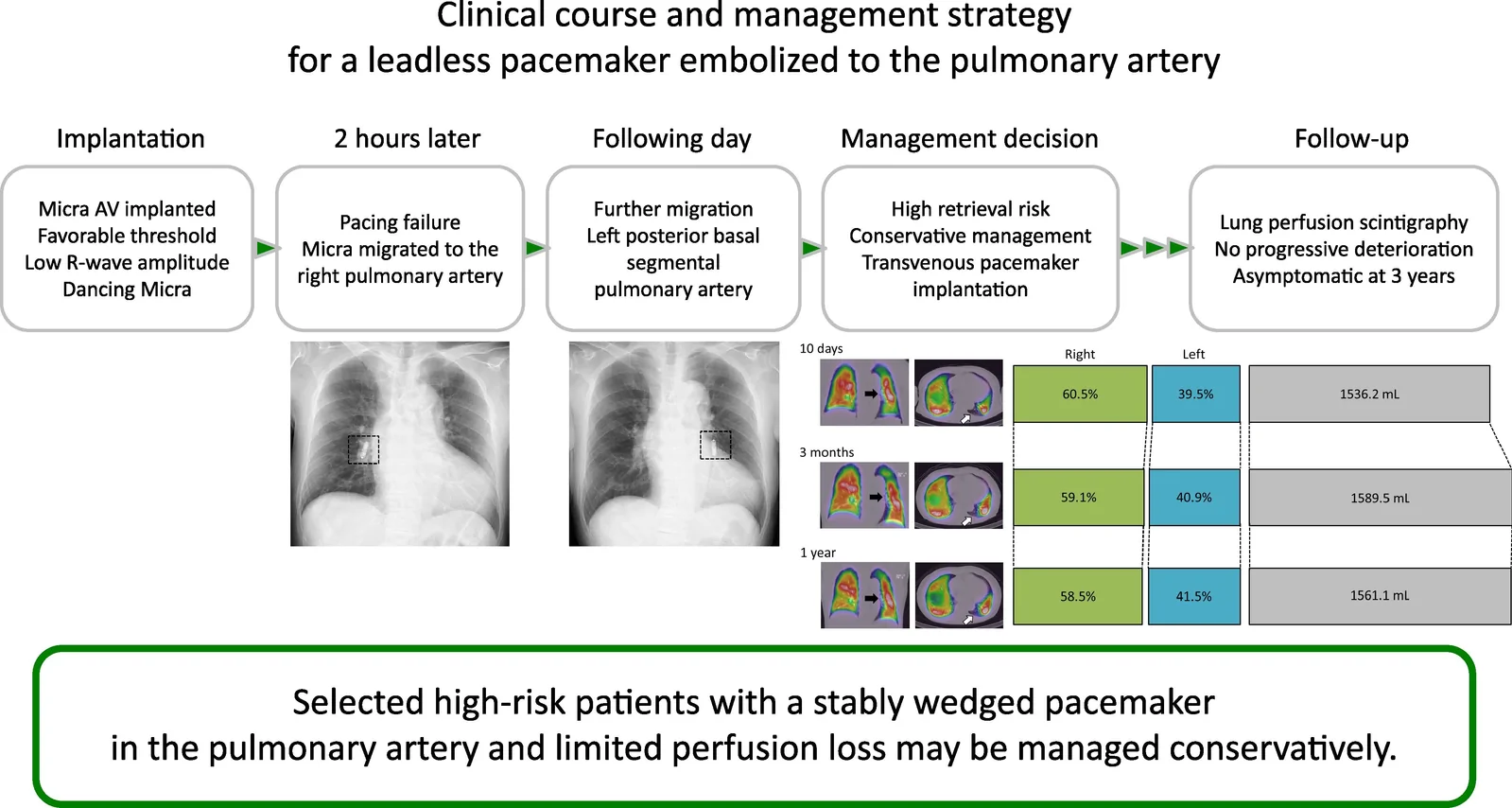


## Case presentation

An 84-year-old woman (height 138 cm, weight 45 kg) presented with fatigue and was found to have complete atrioventricular block with a heart rate of 40 bpm. She had undergone surgery for tongue cancer 5 years earlier, with no evidence of recurrence, and underwent annual follow-up with an otolaryngologist. There was no history of cardiovascular or pulmonary disease, and she was not taking any medications, including antiplatelet or anticoagulant agents, at the time of implantation. Transthoracic echocardiography showed a left atrial diameter of 40 mm, a left ventricular end-diastolic diameter of 38 mm, and a left ventricular ejection fraction of 60%. A mildly elevated B-type natriuretic peptide level of 118 pg/mL was noted (normal range, <18.4 pg/mL).

Although the patient was an elderly woman of small stature, she remained physically active. After discussing the available pacing options, including transvenous and leadless pacemakers, a leadless pacemaker was selected in accordance with her preference. A leadless pacemaker (Micra AV, Medtronic, Minneapolis, MN, USA) was implanted. Because an acceptable pacing threshold could not be obtained, the Micra were deployed in the right ventricular septum on the fifth deployment attempt, and fixation of 2 tines was confirmed by a pull-and-hold test. The pacing threshold was 1.13 V at 0.24 ms, the R-wave amplitude was 2.6 mV, and the impedance was 530 Ω. However, after the tether was cut, the Micra exhibited slight dancing Micra behaviour, suggesting insufficient anchoring.^4^

Two hours later, pacing failure was observed on continuous electrocardiographic monitoring. Chest radiography revealed dislodgement of the leadless pacemaker into the right pulmonary artery (*Figure 1A* and *B*). Considering the risk of pulmonary artery injury, we decided not to attempt retrieval. The following morning, chest radiography (*Figure 1C* and *D*) and computed tomography demonstrated migration of the device from the right pulmonary artery to the left posterior basal segmental pulmonary artery (*Figure 2*). Fortunately, the Micra remained stably positioned at the same site; therefore, a transvenous pacemaker was subsequently implanted. After confirming that the position of the leadless pacemaker remained unchanged and that the transvenous lead was stable for one week, the patient was discharged.

**Figure 1:**
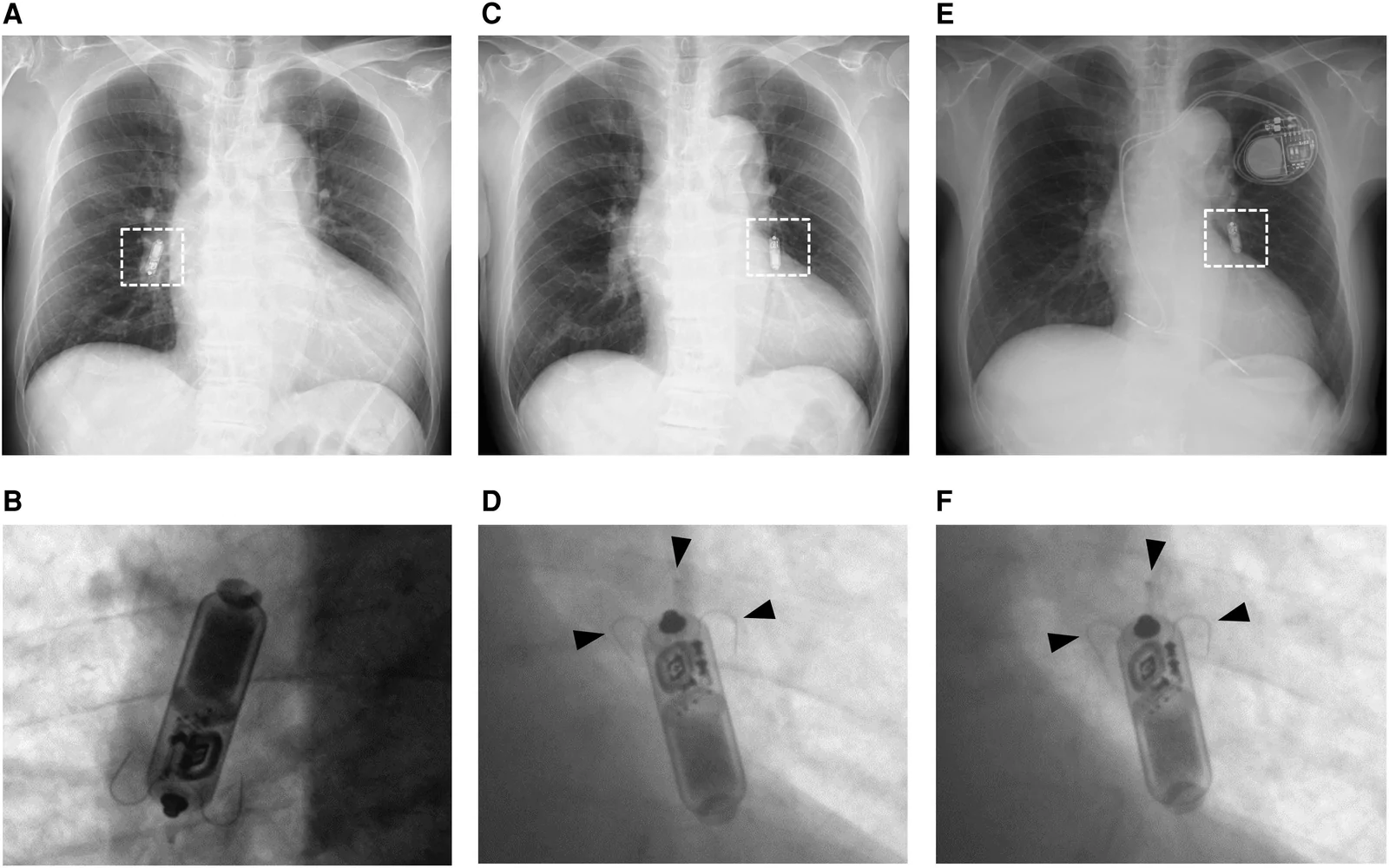
Chest radiographs demonstrate migration of the leadless pacemaker (Micra) into the pulmonary artery (dashed square). Lower panels show magnified views of the dashed squares in the upper images. (*A*) Two hours after implantation, the Micra were located in the right pulmonary artery. (*B*) The distal tip was oriented caudally. (*C*) The following day, it migrated to the left pulmonary artery. (*D*) The tip was oriented cranially. (*E*) One year later, it remained stably positioned in the left pulmonary artery. (*F*) The configuration of the Micra tines (arrowheads) was unchanged compared with the following day.

**Figure 2:**
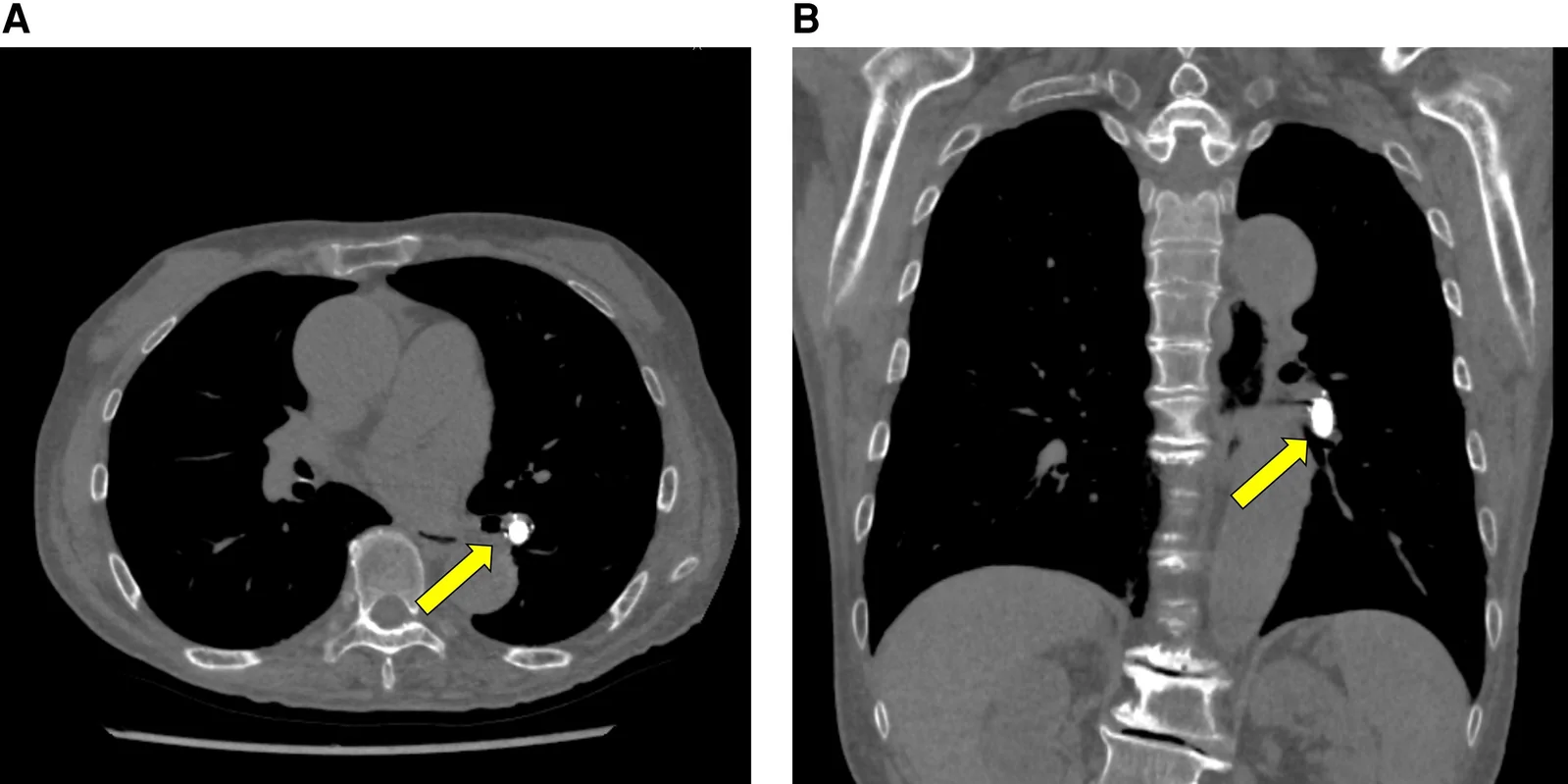
Chest computed tomography images. (*A*) Axial and (*B*) coronal images showing the Micra within the left posterior basal segmental pulmonary artery. Arrows indicate the embolized Micra.

Regular follow-up examinations, including lung perfusion scintigraphy using technetium-99 m macro aggregated albumin (99mTc-MAA), were performed in the outpatient clinic. Pulmonary perfusion scintigraphy performed 10 days after pulmonary embolization showed a right-to-left perfusion distribution of 60.5% vs. 39.5%. At 3 months, the distribution was 59.1% vs. 40.9%, and at 1 year, it was 58.5% vs. 41.5%. Left lung volume was 1536.2 mL at 10 days, 1589.5 mL at 3 months, and 1561.1 mL at 1 year (*Figure 3*). The patient’s condition was considered stable based on the absence of dyspnoea, chest pain, or haemoptysis; the unchanged position and configuration of the device and tines on serial chest radiographs (*Figures 1C–F*); confirmation by computed tomography that the device was wedged in the left posterior basal segmental pulmonary artery (*Figure 2*); and the absence of progressive perfusion impairment or a significant reduction in left lung volume (*Figure 3*). At 3 years of follow-up, the patient remains asymptomatic and can attend outpatient visits independently on foot.

**Figure 3:**
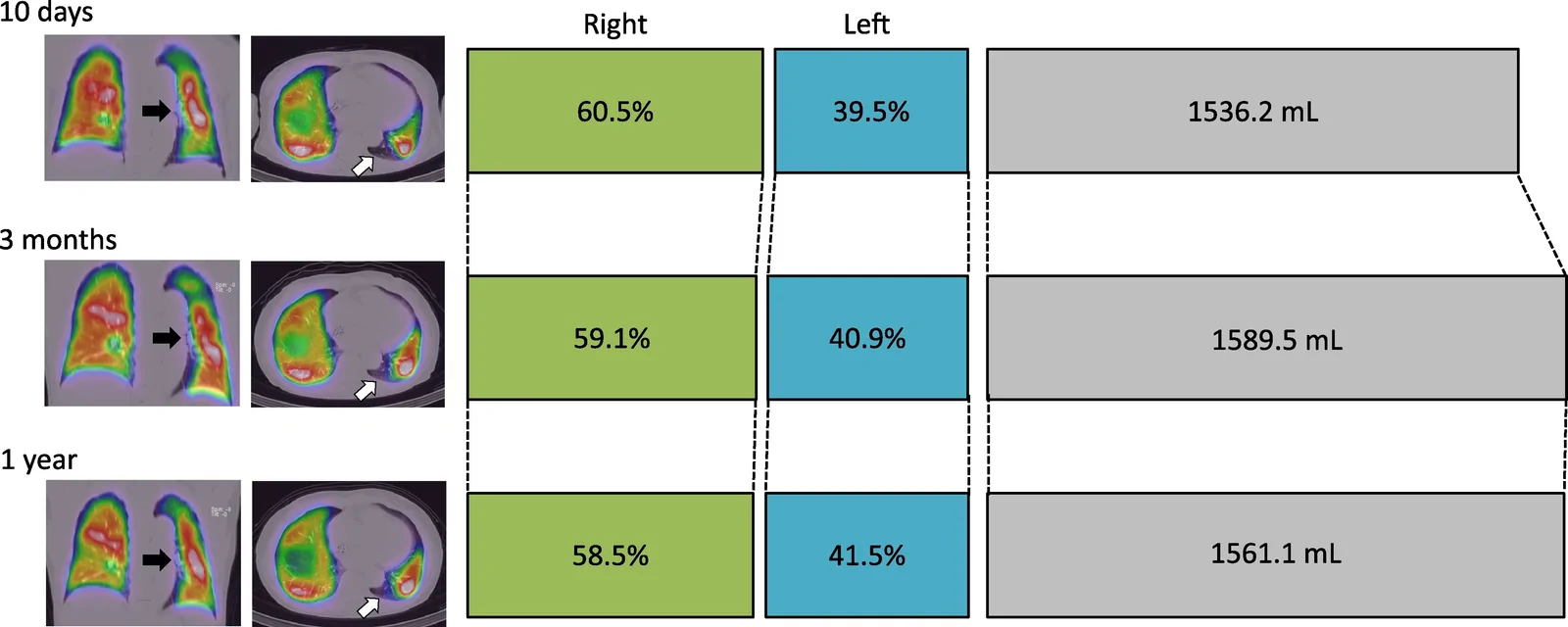
Serial 99mTc-macroaggregated albumin (MAA) lung perfusion scintigraphy after pulmonary embolization of the Micra, showing right-to-left perfusion distribution and left lung volume. Black arrows indicate the embolized Micra, and white arrows indicate regions without 99mTc-MAA uptake. At 10 days, perfusion distribution was 60.5% in the right lung and 39.5% in the left lung. At 3 months, perfusion was 59.1% (right) and 40.9% (left). At 1 year, perfusion was 58.5% (right) and 41.5% (left). Left lung volume was 1536.2 mL at 10 days, 1589.5 mL at 3 months, and 1561.1 mL at 1 year.

## Discussion

Leadless pacemakers were developed to reduce complications associated with traditional transvenous permanent pacemakers, such as pneumothorax, hemothorax, pocket-related complications, and lead dislodgement. According to the Micra Post-Approval Registry, which reflects real-world clinical practice, Micra has demonstrated a sustained high safety profile over long-term follow-up. At 36 months post-implantation, the leadless pacemaker group showed a 53% lower risk of major complications compared with the transvenous pacemaker group (4.1% vs. 8.5%; hazard ratio, 0.47; *P* < 0.001). Major complications, including infection (0.28%) and cardiac effusion or perforation (0.44%), were infrequent. Pulmonary embolization, although extremely rare (0.06%), is a complication unique to leadless pacemakers.^1^ While prompt retrieval of a dislodged leadless pacemaker is generally considered important, retrieval in the setting of pulmonary artery embolization carries potential risks of interference with or injury to the tricuspid valve, chordae tendineae, and other intracardiac structures.^2,3^ In retrospect, greater attention to early indicators of suboptimal fixation during Micra implantation might have been warranted. Although the pacing threshold was favourable at 1.13 V (0.24 ms), the R-wave amplitude was only 2.6 mV in retrospect, and unstable movement known as dancing Micra^4^ was observed after tether release, suggesting inadequate fixation. Although no tine deformation was observed fluoroscopically, repeated deployment attempts may have caused tine damage, and the possibility that such damage contributed to the unstable movement and subsequent embolization cannot be excluded. Performing early retrieval at this stage might have prevented migration into the pulmonary artery.

Leadless pacemakers are more commonly utilized in elderly patients and those with comorbidities compared with transvenous pacemakers, as in the present case. Patients receiving leadless pacemakers therefore constitute a high-risk population with multiple underlying medical conditions.^1^ Furthermore, Micra is not primarily designed for retrieval, and because the actual frequency of retrieval is extremely low, the procedure can be technically challenging at some institutions. The implantation and subsequent management in the present case were performed at a university hospital with five cardiac electrophysiologists, where 120–160 cardiac implantable electronic device procedures and 10–15 transvenous lead extraction procedures are performed annually. After careful consideration, the potential risks associated with retrieval were judged to outweigh its benefits.

In this case, Micra AV was selected for complete atrioventricular block. More recently, active-fixation dual-chamber leadless pacing systems have become available and may allow easier repositioning or retrieval when fixation is inadequate, potentially reducing the risk of embolization. However, it has also been reported that retrieval should be performed with careful attention to the exposed helix if such a device embolizes.^5^ In a mirror-image pattern to our case, a previous report described a leadless pacemaker that migrated into the left pulmonary artery immediately after implantation and became lodged in the right pulmonary artery the following day.^6^ Device stabilization appears to be independent of laterality and instead depends on the pulmonary artery diameter relative to the leadless pacemaker size and whether the tines engage the surrounding tissue. The device in our case likely migrated from the right to the left pulmonary artery through the main pulmonary artery. Serial chest radiography with magnified views of the device and tines is important to confirm device stability (*Figures 1D* and *F*). In the present case, the embolization was limited to a segmental branch of the pulmonary artery, and after carefully weighing the risks associated with leadless pacemaker retrieval, we chose a conservative management strategy without retrieval. The patient remained asymptomatic throughout follow-up. Serial pulmonary perfusion scintigraphy demonstrated reduced perfusion in a portion of the left lung; however, no progression was observed, and there was no significant reduction in lung volume. Previous reports have suggested that a dislodged leadless pacemaker may be harmless if it becomes stably wedged within the pulmonary artery,^6^ and this observation was quantitatively confirmed by pulmonary perfusion scintigraphy in the present patient. In a previous study using 99mTc-MAA pulmonary perfusion scintigraphy, the normal right-to-left lung perfusion ratio in women aged ≥60 years was reported to be 52.9 ± 3.0% for the right lung and 47.1 ± 3.0% for the left lung.^7^ In the present case, marked asymmetry was observed in the acute phase (right 60.5% vs. left 39.5%); however, at 3 months, left lung volume increased by 3.5%, resulting in a reduced perfusion imbalance. At 1 year, the right-to-left perfusion ratio improved to 58.5% and 41.5%, respectively. While many reports^4,5,8–11^ have focused on the technical aspects of leadless pacemaker retrieval, this case suggests that a dislodged leadless pacemaker, if stably wedged within the pulmonary artery, may be managed conservatively without adverse effects on pulmonary perfusion or lung volume and may represent a reasonable alternative strategy in carefully selected high-risk patients. Over a 3-year follow-up period, the patient has remained clinically stable with a favourable course.

Patient perspective: The patient understood the potential risks associated with device retrieval and agreed to conservative management. She was reassured by the absence of symptoms and the stable findings on serial imaging.

## Conclusion

In selected high-risk patients, conservative management of a leadless pacemaker embolized to the pulmonary artery may be feasible when the device is stably wedged. Serial lung perfusion scintigraphy can provide objective evidence of preserved pulmonary function during long-term follow-up.

## Lead author biography


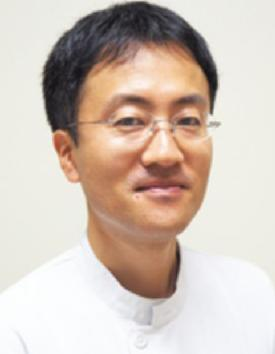


Dr Takanori Arimoto earned his MD and PhD from Yamagata University School of Medicine. He is currently a cardiologist in the Department of Cardiology, Pulmonology, and Nephrology at the same institution. His primary clinical and research interests focus on catheter ablation and device therapy for cardiac arrhythmias.

## Data Availability

The data underlying this article will be made available by the corresponding author upon reasonable request.
